# Increasing the Hardness and Corrosion Resistance of the Surface of CP-Ti by Plasma Electrolytic Nitrocarburising and Polishing

**DOI:** 10.3390/ma16031102

**Published:** 2023-01-27

**Authors:** Sergei Kusmanov, Ivan Tambovskiy, Sergey Silkin, Roman Nikiforov, Roman Belov

**Affiliations:** Department of Chemistry, Institute of Mathematical and Natural Sciences, Kostroma State University, 156005 Kostroma, Russia

**Keywords:** plasma electrolytic treatment, nitricarburising, polishing, PENC, PEP, CP-Ti, surface roughness, microhardness, corrosion resistance

## Abstract

The possibility of increasing the hardness to 1420 HV and the corrosion resistance of the CP-Ti surface using a combined plasma electrolytic treatment consisting in anodic plasma electrolytic nitrocarburising in a solution of ammonia, acetone and ammonium chloride at 900 °C and subsequent plasma electrolytic polishing is shown. The morphology, surface roughness, phase composition, structure and microhardness of the modified layer were studied. The corrosion characteristics of the treated surface were studied through potentiodynamic tests and electrochemical impedance spectroscopy. It has been shown that an increase in the surface roughness has a negative effect on the corrosion resistance. The proposed plasma electrolytic polishing makes it possible to remove the outer porous oxide layer, providing increased corrosion resistance. The highest reduction in the corrosion current density, by 13 times compared to CP-Ti and by two orders compared to a plasma electrolytic nitrocarburising sample, is achieved after plasma electrolytic polishing in a solution of ammonium fluoride (4%) at 300 V for 3 min.

## 1. Introduction

Almost all high-alloy titanium alloys have high corrosion resistance. At the same time, the use of products made of non-alloy or low-alloy materials requires additional surface protection. The increase in corrosion resistance can be carried out by surface modification [1,2]. This will allow the use of cheaper materials, giving the necessary properties only to the contacting part of the product. In addition, it is possible to improve the other properties of the products, for example, surface hardness, which is relevant for titanium alloys, or surface roughness, which is one of the finishing tasks of any surface treatment. One of the methods for the surface modification of metals and alloys is plasma electrolytic treatment. This technology makes it possible to solve a set of tasks for the surface modification of various metal products by combining the diffusion saturation of the surface with atoms of light elements and heat treatment.

The most common type of plasma electrolytic treatment is plasma electrolytic nitrocarburising (PENC), which is implemented with the cathode and anode polarity of the treated samples [3]. At PENC, the surface layer is saturated with nitrogen and carbon atoms at high temperatures, followed by quenching in electrolyte. Various organic compounds can serve as carbon sources during the saturation of titanium alloys, but only ammonia and carbamide (urea) have shown efficiency for nitrogen saturation [4].

The anodic PENC of a Ti6Al4V titanium alloy in a solution containing ammonia, acetone and ammonium chloride leads to the formation of oxide and diffusion layers [5], and after treatment in a solution of carbamide and ammonium chloride, only rutile is detected in the surface layer [6]. According to elemental analysis, the maximum carbon concentration in the surface layer is 2.4% at 800 °C for 5 min of treatment. At 850 °C, the carbon concentration decreases, and at 900 °C, it disappears. The oxygen concentration increases with the increasing temperature and exceeds 8% at 900 °C. In the case of the anodic PENC of an alpha + beta-titanium alloy in a carbamide electrolyte, the surface layer contains titanium oxide TiO_2_ (rutile) and intermetallides Ti_0.8_V_0.2_, Ti_0.75_V_0.25_, MoTi [7]. The modified sample contains an outer oxide layer and a diffusion layer, in which there are beta-phase secretions due to martensitic transformation, the temperature of which is reduced by nitrogen and carbon.

With the PENC of titanium alloys, the hardness of the outer layer increases. The microhardness of commercial pure titanium (CP-Ti) after cathodic PENC reaches 1100 HK at 900 °C [8]. The anodic PENC of Ti6Al4V alloy in carbamide electrolyte at 950 °C provides an increase in the hardness of up to 740 HV [6]. The microhardness of the Ti6Al4V alloy increases to a greater extent after anodic PENC in a solution containing ammonia, acetone and ammonium chloride, reaching up to 940 HV at 850 °C [5]. 

An increase in the surface roughness of titanium alloys is also characteristic of cathodic PENC [9]. Conversely, after anodic PENC, the roughness decreases somewhat [5,10].

The corrosion resistance of CP-Ti in Ringer’s solution is significantly increased by cathodic PENC in various solutions [11]. The best results were obtained with PENC in a triethanolamine solution, where the greatest shift of the corrosion potential in the positive direction, the minimum corrosion current density and the maximum polarization resistance are achieved. An additional increase in the corrosion resistance of CP-Ti is achieved by using pulsed PENC in the same triethanolamine solution [12]. In this case, it is established that the higher the frequency and the lower the fill factor are, the better the corrosion characteristics are. An increase in the frequency of the pulsed PENC of the Ti6Al4V titanium alloy in a solution of formamide (50%) and potassium chloride also contributes to an increase in its corrosion resistance in a solution of 3.5% sodium chloride [9]. Cathodic PENC in a solution of triethanolamine, carbamide and formamide also increases the corrosion resistance of CP-Ti in a solution of Tyrode [13]. After the PENC, the corrosion potential shifts in a positive direction. In addition, tests have shown a good biological compatibility of treated titanium with blood [14].

The formation of an oxide layer during the anodic PENC of titanium alloys does not generally lead to an increase in the corrosion resistance. In addition, the uncontrolled peeling of a part of the oxide layer, particularly at high treatment temperatures, significantly reduces the reproducibility of the results. Therefore, the choice of modes of anodic PENC was carried out in the direction of minimizing the deterioration of the corrosion resistance under the condition of increasing the wear resistance [7,10].

The purpose of this work is to study the possibility of increasing the corrosion resistance of CP-Ti after anodic PENC while maintaining the high hardness of the surface. As a solution to this problem, carrying out subsequent plasma electrolytic polishing (PEP) to remove the outer porous oxide layer and align the surface profile is proposed [15].

## 2. Materials and Methods

### 2.1. Samples Processing

**Figure 1:**
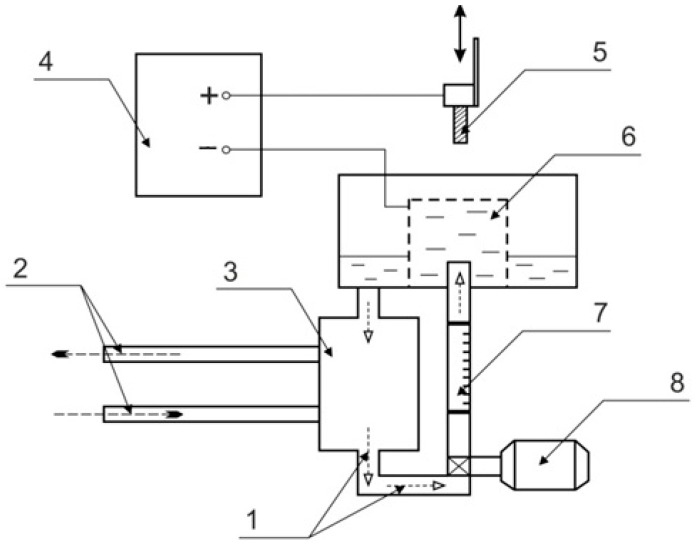
Schematic diagram of setup for anodic PENC and PEP: 1—electrolyte; 2—cold/hot water; 3—heat exchanger; 4—power supply; 5—treated sample; 6—electrolytic cell; 7—flowmeter; 8—pump.

Cylindrical samples (∅ 10 mm × 15 mm) of CP-Ti (99.33 wt.% Ti; 0.25 wt.% Fe; 0.2 wt.% O, 0.1 wt.% Si; 0.07 wt.% C; 0.04 wt.% N; 0.01 wt.% H) were ground with SiC abrasive paper grit size P120 to Ra~1.0 μm and ultrasonically cleaned with acetone. These samples were subjected to duplex surface treatment, which includes sequential anodic PENC and PEP. PENC was carried out in a cylindrical electrolyzer with an axially symmetric electrolyte flow supplied through a nozzle located at the bottom of the electrolyzer (Figure 1). In the upper part of the electrolyzer, the electrolyte was overflowing into the sump and was further pumped through a heat exchanger at a rate of 2.5 L/min, which was measured with 0.4–4 LPM flowmeter (accuracy of ±2.5%). This scheme provides the stabilization of the processing conditions. The solution temperature was measured using a thermosensor, placed at the bottom of the chamber and maintained at 30 ± 2 °C. The samples were connected as the positive output, and the electrolyzer was connected as the negative output of the 15 kW DC power supply. An aqueous solution of ammonia (5 wt.%), acetone (5 wt.%) and ammonium chloride (10 wt.%) was used as the working electrolyte.

After switching the voltage to 200 V, the samples were immersed in the electrolyte at a speed of 1–2 mm/s. If the rate of immersion was slow, a vapor-gaseous envelope easily formed on an initially small surface area of the sample near the electrolyte surface and extended further across the sample as it submerged. Once the sample was immersed at a depth equal to its height, the voltage was changed to 165, 180, 200, and 235 V in order to reach the prescribed sample’s temperature, 750, 800, 850, and 900 °C. The sample temperature was measured with a K-type thermocouple (Termoelement, Moscow, Russia) with multimeter APPA109N (accuracy to 3% over a temperature range of 400–1000 °C). The thermocouple was fixed in a hole made in the samples at a distance of 2 mm from the sample’s bottom. The treatment time was 5 min and, after PENC, the samples were quenched in the electrolyte (hardening).

Following PENC, the samples were subjected to subsequent PEP in the same electrolyzer and in different solutions: ammonium sulfate (3%) at a temperature of 80 °C; ammonium chloride (1%) at a temperature of 80 °C; and ammonium fluoride (4%) at a temperature of 90 °C, with varying voltage and duration. The immersion depth of the upper end was 5 mm. A Teflon bushing was used to prevent the electrolyte from splashing and dissolving the part of the anodic current lead that was submerged in the electrolyte during PEP. The electrolyte was pumped through a heat exchanger at a flow rate of 1 L/min.

### 2.2. Study of the Surface Morphology and Microstructure

The Micromed MET (Observing devices, St. Petersburg, Russia) optical metallographic microscope with digital image visualization served to study the surface morphology and microstructure of cross-section of the CP-Ti samples.

### 2.3. The Microhardness Measurement

The microhardness of the cross sections of the treatment sample was measured using a Vickers microhardness tester (Falcon 503, Innovatest Europe BV, Maastricht, The Netherlands) under a 0.1-N load. According to 5 measurements, the average value of microhardness was found.

### 2.4. The Surface Roughness and Weight of Samples Measurement

The surface roughness was measured with a TR-200 profilometer (Beijing TIME High Technology Ltd., Beijing, China). According to 10 measurements, the average value of the roughness indicators was found.

The change in the weight of the samples was determined on a CitizonCY224C electronic analytical balance (ACZET (Citizen Scale), India) with an accuracy of ±0.0001 g, after washing the samples with distilled water to remove traces of salts and subsequently drying.

### 2.5. Study of the Phase Composition

X-ray diffraction (XRD) analysis was used to determine the phase composition of the samples. The XRD patterns were obtained by PANalytical Empyrean X-ray diffractometer (Malvern Panalytical, Malvern, UK) with CoKα radiation by a simple scanning mechanism in the theta-2theta-mode with a step of 0.026° and a scanning rate of 4.5°/min. Phase composition analysis was performed using the PANalytical High Score Plus software, software [16] and the ICCD PDF-2 and COD databases [17].

### 2.6. Study of the Corrosion Resistance

The corrosion resistance of the samples was determined by potentiodynamic polarization curves using a Biologic SP-150 potentiostat-galvanostat (Biologic Science Instruments, France) with a standard three-electrode cell in Ringer’s solution (8.6 g/L NaCl, 0.3 g/L KCl, 0.25 g/L CaCl_2_), at a sweep rate of 1 mV/s. Before the corrosion testing, the samples were cleaned with acetone in the ultrasonic bath for 5 min, then washed with distilled water and dried until their weight stabilized. Further, each samples’ surface was insulated by a dielectric mask with a circular aperture with a 0.132 mm^2^ area located at the distance of 2 mm from the lower edge of the sample. Graphite was used as the auxiliary electrode. The saturated silver chloride electrode served as the reference one. The working electrode (sample) was kept in the Ringer’s solution for 60 min before testing to steady the constant value of the corrosion potential. The corrosion current density was determined by Tafel’s extrapolation of the polarization curves using EC-Lab software (v.11.43).

The mechanism of the corrosion processes was studied by electrochemical impedance spectroscopy after an hour of exposure to electrolyte at an open circuit potential of ±10 mV, in the frequency range 100 MHz–100 kHz. The processing of the received data was carried out using the Zview software (v.2), based on the selected equivalent scheme. Due to the very high corrosion resistances, which means very low currents and the possibility of influencing the accuracy of the interference measurements, all of the corrosion (including impedance) measurements were carried out in a Faraday cell.

## 3. Results

### 3.1. Morphology and Roughness of the Surface after PENC

**Figure 2:**
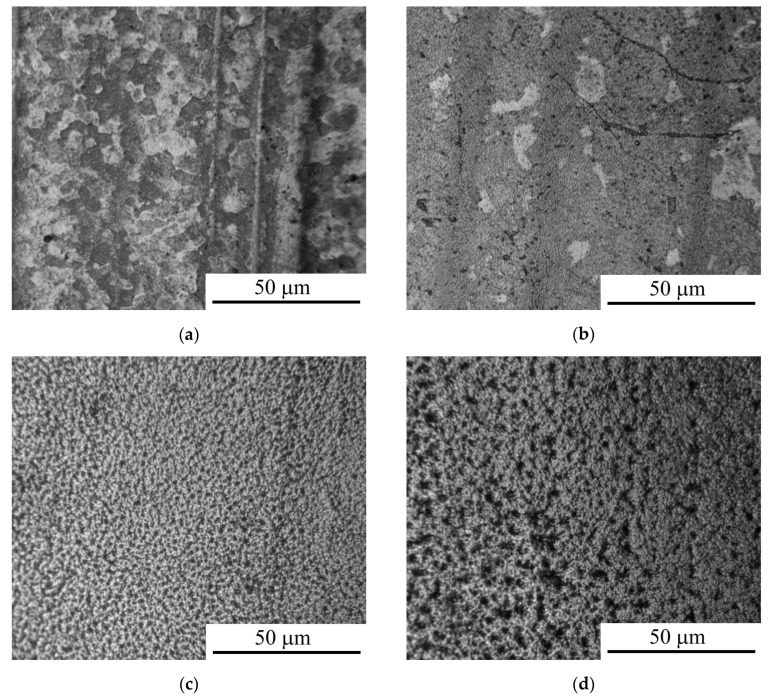
Morphology of the CP-Ti surface after anodic PENC at different treatment temperature: (**a**) 750 °C; (**b**) 800 °C; (**c**) 850 °C; (**d**) 900 °C.

The analysis of the changes in the morphology and roughness of the surface of CP-Ti after anodic PENC showed that with an increase in the treatment temperature to 850 °C, the surface relief is smoothed (Figure 2), which is reflected in a decrease in roughness by almost two times (Table 1). With a subsequent increase in the temperature to 900 °C, the surface is uniformly detected with the resulting irregularities (Figure 2d) leading to an increase in roughness.

### 3.2. Phase Composition, Structure and Microhardness of the Surface Layer after PENC

**Figure 3:**
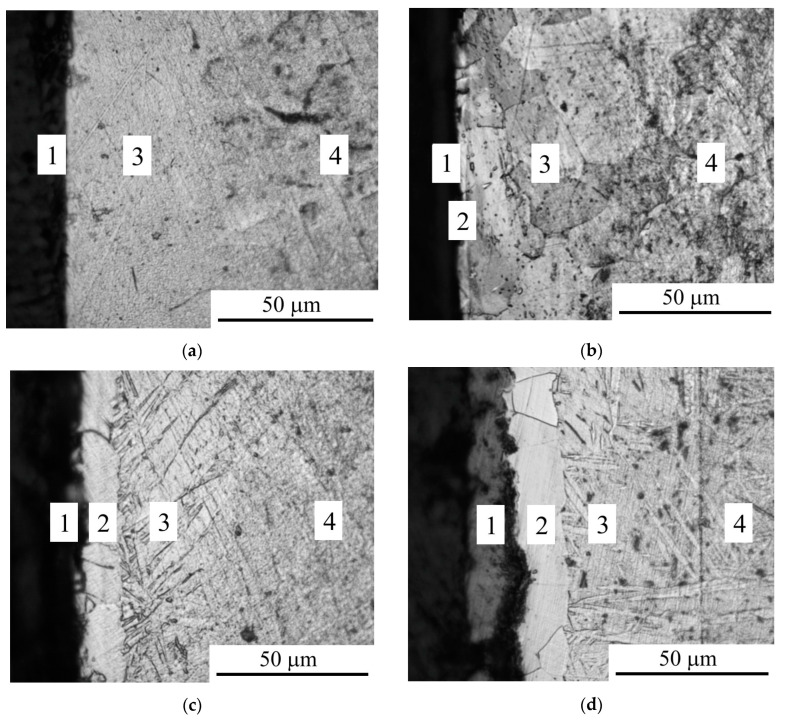
Microstructure of cross-section of the CP-Ti surface after anodic PENC at different treatment temperature: (**a**) 750 °C; (**b**) 800 °C; (**c**) 850 °C; (**d**) 900 °C. 1—oxide layer; 2—nitrided layer; 3—diffusion layer (N and C solid solution); 4—initial structure.

**Figure 4:**
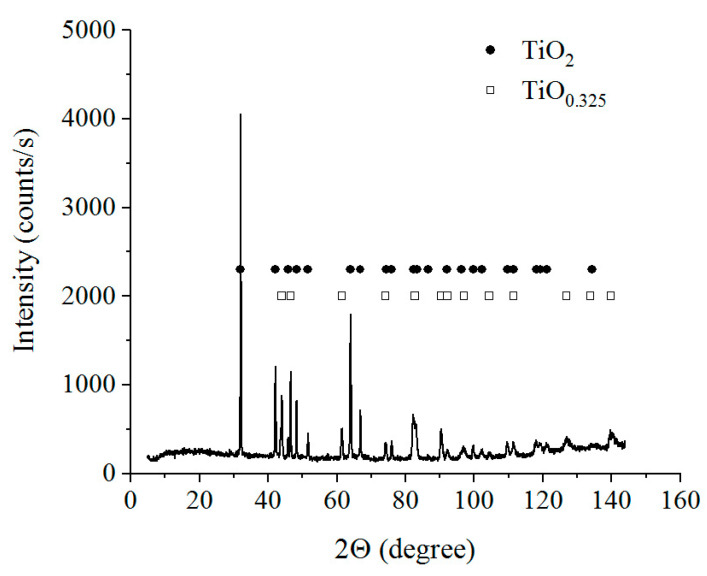
X-ray diffraction patterns of the CP-Ti surface layer after anodic PENC at 900 °C.

**Figure 5:**
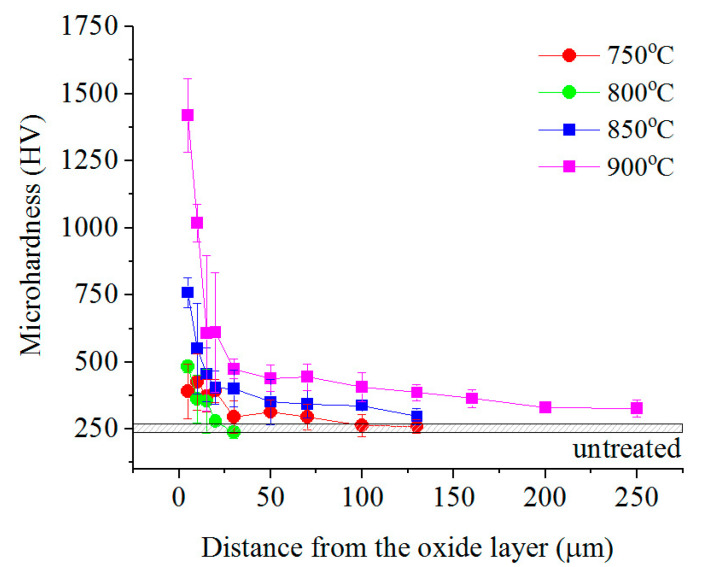
Microhardness distribution in the surface layer after anodic PENC at different treatment temperature.

According to a metallographic analysis, the anodic PENC of the titanium alloy surface leads to the formation of an oxide layer and a diffusion layer—a solid solution of carbon and nitrogen in the initial structure of the alloy (Figure 3). According to X-ray analysis, the oxide layer consists mainly of anatase (TiO_2_) and a solid solution of the composition TiO_0.325_ (Figure 4). Starting from 850 °C, a nitrided layer is detected on the surface, growing with an increase in the treatment temperature to 20 microns. Similarly, with an increase in the treatment temperature, the microhardness increases, which is reached after 900 °C to 1280 HV, exceeding the microhardness of the initial structure by five times (Figure 5).

### 3.3. Morphology and Roughness of the Surface after PEP

Subsequent PEP was subjected to the samples after PENC at 900 °C, which have high surface layer hardness values. The results of profilometric studies have shown that after PEP in a sulfate electrolyte, the smallest removal of material occurs at a rate equal to (1.3 ± 0.2) mg/min, and a slight increase in roughness within the measurement error, without revealing dependencies on the voltage and duration of PEP (Table 2).

When using a chloride electrolyte, a significant increase in the roughness is observed in proportion to the duration of polishing, regardless of the value of the applied voltage at a higher material removal rate equal to (7.1 ± 1.0) mg/min (Table 3).

**Figure 6:**
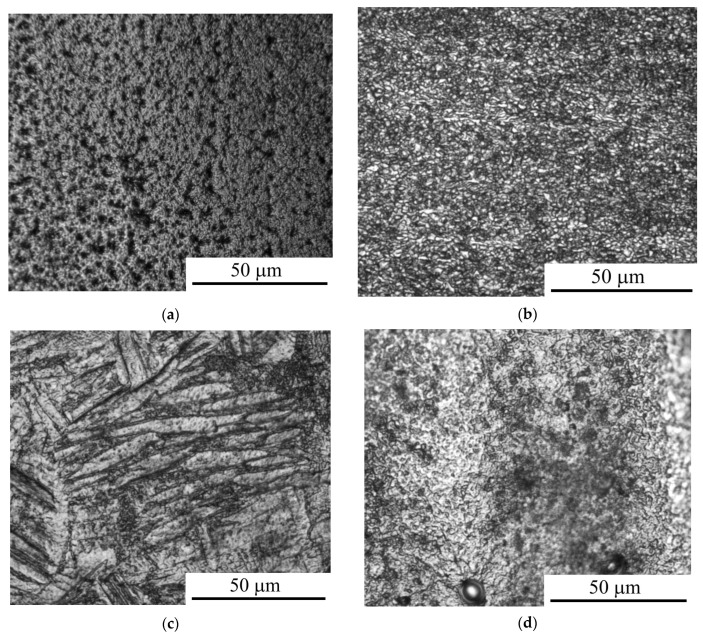
Morphology of the CP-Ti surface after PENC at 900 °C (**a**) and subsequent PEP in a solution of ammonium fluoride (4%) during 5 min at different voltage: (**b**) 275 V; (**c**) 300 V; (**d**) 325 V.

The opposite results were obtained when using a fluoride electrolyte. With an increase in the duration of the PEP, the roughness decreases and, in the case of polishing at 275 and 300 V for more than 3 min, its value becomes lower than the initial value for the nitrocarburised surface (Table 4). According to the morphological analysis, this leads to a uniform removal of the oxide layer (Figure 6). The rate of material loss is even higher, being equal to (16.8 ± 2.5) mg/min.

### 3.4. Corrosion Properties of the Surface after PENC and PEP

**Figure 7:**
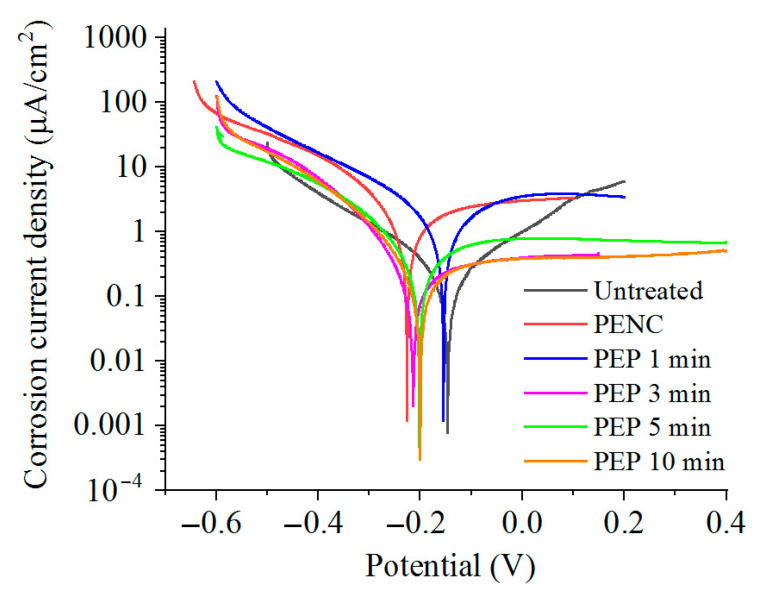
Potentiodynamic polarization curves of the CP-Ti samples before and after anodic PENC at 900 °C and subsequent PEP in a solution of ammonium sulfate (3%) at 300 V for different time.

**Figure 8:**
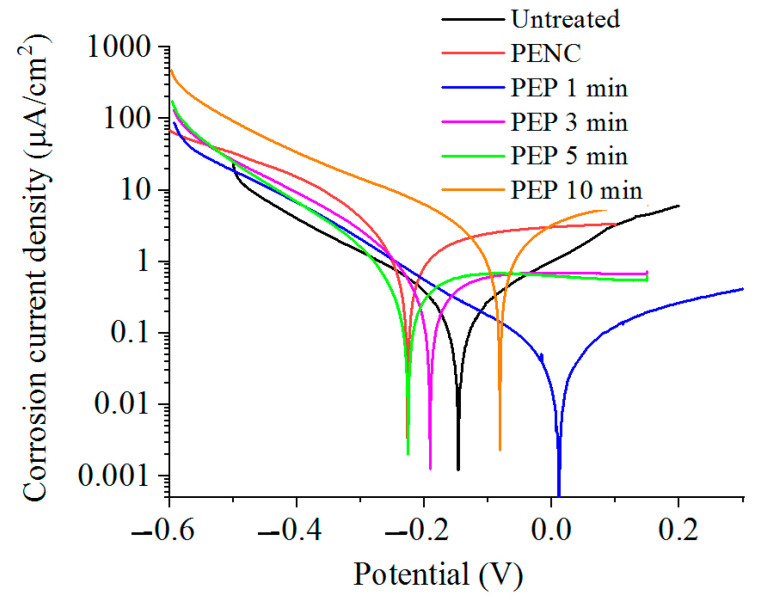
Potentiodynamic polarization curves of the CP-Ti samples before and after anodic PENC at 900 °C and subsequent PEP in a solution of ammonium chloride (1%) at 300 V for different time.

**Figure 9:**
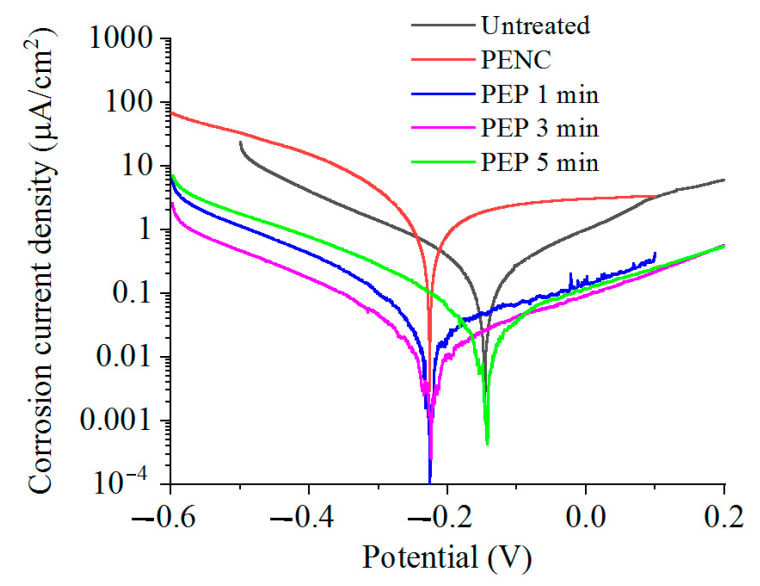
Potentiodynamic polarization curves of the CP-Ti samples before and after anodic PENC at 900 °C and subsequent PEP in a solution of ammonium fluoride (4%) at 300 V for different time.

The corrosion tests in the Ringer’s solution showed an increase in the corrosion current density after the anodic PENC (Table 1). An increase in the treatment temperature similarly leads to an increase in the corrosion current. Subsequent PEP, in a solution of ammonium sulfate (3%) at 275 V from 3 to 10 min and at 300 V for 3 min, reduced the corrosion current density to 1.5 times lower than that of the untreated sample (Table 2, Figure 7). PEP in a solution of ammonium chloride (1%) at 275 V for 3 min and 300 V for 1 min allowed the corrosion resistance to be improved further. The corrosion current density decreased by 1.6 and 5.4 times, respectively, compared with the untreated sample, while in the last and only case, a positive value of the corrosion potential was observed (Table 3, Figure 8). A significant improvement in the corrosion resistance was observed after PEP in an ammonium fluoride solution (4%) (Table 4, Figure 9). A decrease in the corrosion current density occurred in all modes, except PEP at 275 V for 1 and 3 min, and reaches up to 13.5 times compared with the untreated and 101.5 times with the PENC samples.

**Figure 10:**
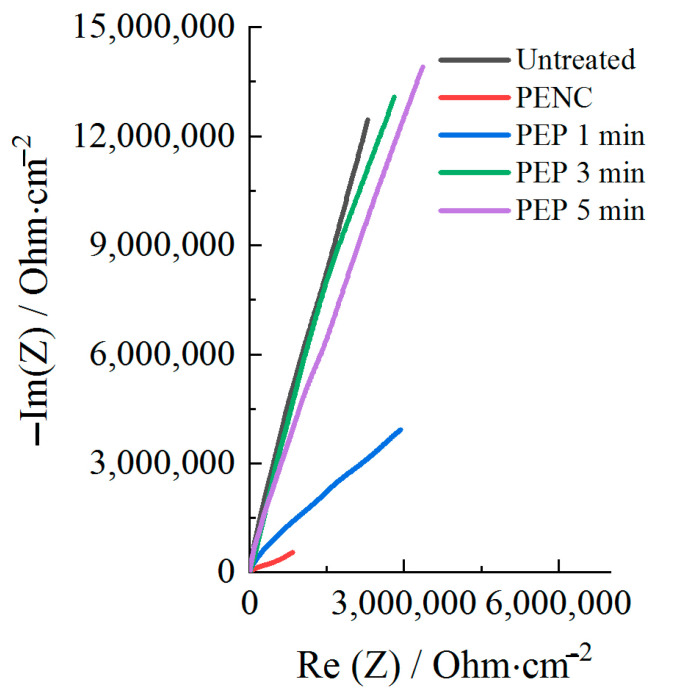
Nyquist plots of the CP-Ti samples before and after anodic PENC at 900 °C and subsequent PEP in a solution of ammonium fluoride (4%) at 300 V for different time.

**Figure 11:**
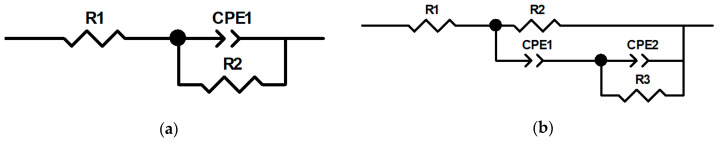
Equivalent electrical circuit model used for impedance data fitting: (**a**) for the untreated samples and the PEP for 3 and 5 min samples; (**b**) for the PENC and PEP for 1 min treated samples.

Figure 10 shows the results of the electrochemical impedance spectroscopy of the untreated and treated samples after anodic PENC and PEP in a solution of ammonium fluoride. In order to describe the electrochemical interface of the materials, different equivalent circuits were analyzed to obtain the best fit, and conceivable equivalent circuit models of the untreated and treated samples are shown in Figure 11. In the equivalent circuit model, R1 represents the solution resistance; R2 and CPE1 correspond to the passive layer (including diffusion layer after PENC) of the CP-Ti; R3 and CPE2 represent the outer porous oxide layer. The equivalent circuit model for the untreated samples and the PEP for 3 and 5 min samples consisted of a single time constant (CPE1) with a resistor (R2) in parallel and the solution resistance (R1) in series, as shown in Figure 11a. The equivalent circuit model for the PENC and PEP for 1 min treated samples consisted of two time constants, namely CPE1 with a resistor (R2) in parallel, and CPE2 a resistor (R3) in parallel and the solution resistance (R1) in series, as shown in Figure 11b. Similar circuits have been reported for the plasma electrolytic oxidation of CP-Ti [18,19]. The results of the calculations of the elements of the equivalent schemes from the experimental data are presented in Table 5.

The results show that the resistance of the substrate and diffusion layer (R2) was higher than the corresponding value of the outer porous oxide layer (R3), as reported in Table 5. According to the values of R2 and R3, the highest corrosion resistance is shown in the CP-Ti sample after PENC and subsequent PEP for 3 min, when the outer porous oxide layer is removed.

## 4. Discussion

The morphology of the surface of CP-Ti in the process of anodic PENC is determined by the simultaneous action of oxidation with the formation of an oxide layer consisting of anatase (TiO_2_) and a solid solution of the composition TiO_0.325_, and anodic dissolution. At treatment temperatures up to 850 °C, anodic dissolution prevails over oxidation, leading to a decrease in the roughness. With an increase in the temperature of PENC, oxidation prevails over anodic dissolution, which is reflected in an increase in the roughness. Similar patterns were found in the PENC of low-carbon steel in the same electrolyte [20], which indicates a similar mechanism of processes.

Despite the inhibitory effect of oxidation on diffusion [21] with an increase in the PENC temperature, the diffusion layers’ thickness and hardness increase. Therefore, it is more expedient to carry out PENC at high temperatures to achieve high hardness of the surface layer. At the same time, at high temperatures, the observed development of the surface relief negatively affects the corrosion characteristics. The shown effect of the PENC temperature on the hardness of the diffusion layers is similar to what was revealed during the PENC of steels [20,22] and anodic plasma electrolytic nitriding of titanium alloys [23] and steels [24]. Thus, the processes of diffusion, oxidation and anodic dissolution during the PENC of titanium alloys and steels have similar patterns.

The proposed solution to the problem, by removing the outer porous oxide layer using PEP, showed the possibility of reducing the surface roughness. The influence of the nature of the electrolyte anion on the polishing efficiency is shown. The use of an oxygen-containing sulfate anion leads to minimal material removal due to the additional passivation of the surface. With PEP in chloride and fluoride electrolyte, the outer oxide layer is removed at a higher rate, in proportion to the activity of anions. When using a chloride electrolyte, the development of relief irregularities and an increase in the roughness is observed due to the etching of fewer solid sections of the oxide layer in proportion to the PEP time. The use of a fluoride electrolyte leads to the uniform and nearly complete removal of the outer oxide layer until the metallic texture is revealed due to the dissolution of titanium oxide by the hydrofluoric acid formed during the hydrolysis of ammonium fluoride.

According to the data of the potentiodynamic tests, there is a correlation between the increase in the corrosion resistance and the decrease in the surface roughness. The observed correlation of the decrease in the corrosion current density allows us to judge the formation of a homogeneous corrosion-resistant surface. In the case of using a sulfate electrolyte, the pores and irregularities are filled with formed oxides under the action of oxygen-containing anions. This leads to a decrease in the corrosion current density in proportion to the PEP time. A significant effect is observed, with the complete and uniform removal of the outer porous oxide layer, which is typical when using a fluoride electrolyte. According to the electrochemical impedance spectroscopy, the oxide coating has a low resistance, and its removal makes it possible to increase the corrosion resistance, where the diffusion layer acts as a barrier to charge transfer. A low CPE1 value for a PENC sample (Table 5) means that the oxide layer has large pores and low permittivity. The best results, according to potentiodynamic tests and electrochemical impedance spectroscopy, were shown on a PENC sample after PEP in a fluoride electrolyte at 300 V for 3 min. With a shorter PEP time, the oxide layer does not completely dissolve, and after longer treatment, the diffusion layer dissolves, which leads to a decrease in the resistance. This shows the positive effect of the diffusion layer and the negative effect of the porous oxide layer on corrosion resistance.

## 5. Conclusions

1. It is difficult to increase the corrosion resistance of titanium alloys by chemical-thermal hardening because the accompanying occurrence of defects in the surface structure and the development of surface morphology often worsen the corrosion resistance. This paper shows the possibility of simultaneously increasing the hardness and corrosion resistance of the CP-Ti surface using a combined plasma electrolytic treatment consisting in anodic PENC and subsequent PEP. This duplex technology is proposed for the first time for processing titanium alloys, which determines the novelty of this study.
2. It has been found that the increase in the hardness is achieved through the diffusion saturation of the surface with nitrogen and carbon, which increases with the increasing temperature and reaches up to 1420 HV at PENC in a solution of ammonia (5%), acetone (5%) and ammonium chloride (10%) at 900 °C for 5 min.
3. It is revealed that the competing influence of high-temperature oxidation with the formation of an oxide layer and anodic dissolution determines the morphology and roughness of the surface. At the same time, the negative effect of a developed surface with a high roughness on the corrosion resistance is shown.
4. The paper proposes a solution to the problem of reducing the surface roughness through subsequent polishing in an ammonium fluoride solution, which is accompanied by the removal of the outer porous oxide layer.
5. The electrochemical impedance spectroscopy method shows the positive effect of the diffusion layer and the negative effect of the porous oxide layer on the corrosion resistance. The highest reduction in the corrosion current density, by 13 times compared to CP-Ti and by 2 orders compared to a PENC sample, is achieved after PEP in an ammonium fluoride solution (4%) at 300 V for 3 min. Under these conditions, only the outer porous oxide layer is removed and corrosion resistance is provided by the action of the diffusion layer.

## Figures and Tables

**Table 1:** Values of surface roughness, corrosion current density and corrosion potential of CP-Ti samples after anodic PENC at different temperatures.

PENC Temperature (°C)	Surface Roughness Ra (μm)	Corrosion Current Density (μA/cm^2^)	Corrosion Potential (V)
Untreated	1.00 ± 0.10	0.27	–0.147
750	0.61 ± 0.15	0.83	–0.225
800	0.57 ± 0.14	0.68	–0.200
850	0.55 ± 0.14	2.08	–0.145
900	0.80 ± 0.12	2.03	–0.225

**Table 2:** Values of weight loss, surface roughness, corrosion current density and corrosion potential of CP-Ti samples after anodic PENC at 900 °C and subsequent PEP in a solution of ammonium sulfate (3%).

Voltage (V)	PEP Time (min)	Weight Loss of the Sample (mg)	Surface Roughness Ra (μm)	Corrosion Current Density (μA/cm^2^)	Corrosion Potential (V)
Untreated			1.00 ± 0.10	0.27	−0.147
PENC at 900 °C			0.80 ± 0.12	2.03	−0.225
275	1	1.4	0.86 ± 0.10	1.66	−0.246
	3	4.0	0.89 ± 0.07	0.22	−0.234
	5	4.7	0.89 ± 0.08	0.22	−0.148
	10	6.4	1.09 ± 0.12	0.18	−0.069
300	1	2.8	0.87 ± 0.07	1.53	−0.154
	3	4.7	0.90 ± 0.08	0.23	−0.198
	5	6.5	0.89 ± 0.13	0.48	−0.199
	10	9.6	0.85 ± 0.06	0.31	−0.201
325	1	3.9	0.94 ± 0.04	1.84	−0.229
	3	4.3	0.92 ± 0.10	2.83	−0.195
	5	7.1	0.87 ± 0.09	0.61	−0.202
	10	9.9	0.85 ± 0.06	0.64	−0.195

**Table 3:** Values of weight loss, surface roughness, corrosion current density and corrosion potential of CP-Ti samples after anodic PENC at 900 °C and subsequent PEP in a solution of ammonium chloride (1%).

Voltage (V)	PEP Time (min)	Weight Loss of the Sample (mg)	Surface Roughness Ra (μm)	Corrosion Current Density (μA/cm^2^)	Corrosion Potential (V)
Untreated			1.00 ± 0.10	0.27	−0.147
PENC at 900 °C			0.80 ± 0.12	2.03	−0.225
275	1	12.7	1.69 ± 0.50	0.29	−0.214
	3	25.2	3.02 ± 0.56	0.17	−0.051
	5	35.2	5.52 ± 1.08	4.82	−0.131
	10	64.9	7.22 ± 1.05	14.05	−0.236
300	1	13.0	1.51 ± 0.18	0.05	0.011
	3	20.9	3.11 ± 0.47	0.64	−0.190
	5	28.3	4.21 ± 0.34	0.64	−0.224
	10	68.1	6.39 ± 0.68	2.17	−0.081
325	1	12.0	1.50 ± 0.14	0.31	−0.204
	3	26.7	3.20 ± 0.41	0.47	−0.239
	5	34.0	4.25 ± 0.47	0.64	−0.115
	10	70.8	5.20 ± 0.76	4.45	−0.039

**Table 4:** Values of weight loss, surface roughness, corrosion current density and corrosion potential of CP-Ti samples after anodic PENC at 900 °C and subsequent PEP in a solution of ammonium fluoride (4%).

Voltage (V)	PEP Time (min)	Weight Loss of the Sample (mg)	Surface Roughness Ra (μm)	Corrosion Current Density (μA/cm^2^)	Corrosion Potential (V)
Untreated			1.00 ± 0.10	0.27	−0.147
PENC at 900 °C			0.80 ± 0.12	2.03	−0.225
275	1	17.4	0.95 ± 0.07	0.58	−0.077
	3	42.9	0.61 ± 0.05	0.52	−0.151
	5	67.1	0.52 ± 0.08	0.02	−0.258
300	1	27.4	1.01 ± 0.05	0.05	−0.225
	3	52.5	0.75 ± 0.02	0.02	−0.223
	5	88.2	0.76 ± 0.02	0.06	−0.144
325	1	20.3	1.02 ± 0.02	0.23	−0.166
	3	29.6	0.99 ± 0.04	0.24	−0.170
	5	37.1	0.81 ± 0.01	0.08	−0.191

**Table 5:** Various electrical parameter values obtained after equivalent circuit fitting.

Sample	R1 (Ω)	CPE1 (μmF/cm^2^)	*n*	R2 (M Ω/cm^2^)	CPE2 (μmF/cm^2^)	*n*	R3 (M Ω/cm^2^)
Untreated	65.3	29.7	0.93	133	-	-	-
PENC	67.0	1.37	0.70	1.89	39.5	0.71	0.31
PEP 1 min	60.6	70.7	0.94	13.7	53.2	0.83	1.41
PEP 3 min	64.5	28.5	0.84	1120	-	-	-
PEP 5 min	60.4	23.7	0.93	66.5	-	--	-

## Data Availability

Not applicable.
